# EHMTI-0250. General practitioners’ experiences with brief intervention for medication-overuse headache: a qualitative study

**DOI:** 10.1186/1129-2377-15-S1-C32

**Published:** 2014-09-18

**Authors:** ES Kristoffersen, C Lundqvist, JC Frich

**Affiliations:** 1 Department of General Practice, University of Oslo, Oslo, Norway; 2 Research Centre, Akershus University Hospital, Lørenskog, Norway; 3 Institute of Health and Society, University of Oslo, Oslo, Norway

## Introduction

Medication-overuse headache (MOH) is common in the general population, and the majority of sufferers are managed in primary health care. Brief Intervention (BI) has been used as a motivational technique for patients with drug and alcohol overuse, and may a have role in the treatment of MOH.

## Aim

To explore GPs’ experiences using BI in the management of patients with MOH.

## Method

Qualitative study in Norwegian general practice. Data was collected through four focus group interviews with 22 GPs who participated in an intervention study on BI for MOH. We used systematic text condensation to analyse transcripts from the focus group interviews.

## Results

The GPs experienced challenges when trying to convince patients that the medication they used to treat and prevent headache could cause headache, but labelling MOH as a diagnosis opened up space for action. GPs were able to use BI within the scope of a regular consultation, and they thought that the structured approach had a potential to change patients’ views about their condition and medication use. Being diagnosed with medication overuse could bring about feelings of guilt in patients, and GPs emphasised that a good alliance with the patient was necessary for successful change using BI to manage MOH.

## Conclusion

GPs experience BI as a feasible strategy to treat MOH, and the technique relies on a good alliance between the doctor and patient. When using BI, GPs must be prepared to counter patients’ misconceptions about medication used for headache.

No conflict of interest.

